# Evaluation of Environmental Contamination and Estimated Radiation Exposure Dose Rates among Residents Immediately after Returning Home to Tomioka Town, Fukushima Prefecture

**DOI:** 10.3390/ijerph16091481

**Published:** 2019-04-26

**Authors:** Masahiko Matsuo, Yasuyuki Taira, Makiko Orita, Yumiko Yamada, Juichi Ide, Shunichi Yamashita, Noboru Takamura

**Affiliations:** 1 Department of Global Health, Medicine and Welfare, Atomic Bomb Disease Institute, Nagasaki University Graduate School of Biomedical Sciences, Nagasaki, Nagasaki Prefecture 852-8523, Japan; bb55b16008@ms.nagasaki-u.ac.jp (M.M.); orita@nagasaki-u.ac.jp (M.O.); yumiko@nagasaki-u.ac.jp (Y.Y.); takamura@nagasaki-u.ac.jp (N.T.); 2 Nuclear Safety Research Association, Tokyo 105-0004, Japan; j-ide@fukushima-power.com; 3 Special Advisor to the President, Nagasaki University, Nagasaki, Nagasaki Prefecture 852-8521, Japan; shun@nagasaki-u.ac.jp

**Keywords:** air dose rate, difficult-to-return zone, evacuation order-lifted areas, effective dose rate, external exposure risk, Fukushima Daiichi Nuclear Power Station accident, living space, radiocesium, surface soil, Tomioka town

## Abstract

On 1 April 2017, six years have passed since the Fukushima Daiichi Nuclear Power Station (FDNPS) accident, and the Japanese government declared that some residents who lived in Tomioka Town, Fukushima Prefecture could return to their homes. We evaluated environmental contamination and radiation exposure dose rates due to artificial radionuclides in the livelihood zone of residents (living space such as housing sites), including a restricted area located within a 10-km radius from the FDNPS, immediately after residents had returned home in Tomioka town. In areas where the evacuation orders had been lifted, the median air dose rates were 0.20 μSv/h indoors and 0.26 μSv/h outdoors, and the radiation exposure dose rate was 1.6 mSv/y. By contrast, in the “difficult-to-return zone,” the median air dose rate was 2.3 μSv/h (20 mSv/y) outdoors. Moreover, the dose-forming artificial radionuclides (radiocesium) in the surface soil were 0.018 μSv/h (0.17 mSv/y) in the evacuation order-lifted areas and 0.73 μSv/h (6.4 mSv/y) in the difficult-to-return zone. These findings indicate that current concentrations of artificial radionuclides in soil samples have been decreasing in the evacuation order-lifted areas of Tomioka town; however, a significant external exposure risk still exists in the difficult-to-return zone. The case of Tomioka town is expected to be the first reconstruction model including the difficult-to-return zone.

## 1. Introduction

More than eight years have passed since 11 March 2011, the date of the 9.0-magnitude Great East Japan Earthquake, subsequent tsunami, and disaster at the Fukushima Daiichi Nuclear Power Station (FDNPS), which is operated by the Tokyo Electric Power Company. Various radionuclides were released from the FDNPS into the atmosphere, eventually being deposited on land and at sea in the surrounding areas [1]. The estimated total amount of iodine-131 (^131^I) released ranged from about 100–500 petabecquerel (PBq), and that of cesium-137 (^137^Cs) was generally in the range of 6–20 PBq [2]. For perspective, the estimated releases of ^131^I and ^137^Cs from the FDNPS were about 10% and 20%, respectively, of those estimated for the Chernobyl accident [2]. Although much of the released radionuclides was dispersed over the Pacific Ocean, a fraction was dispersed over the eastern mainland of Japan; these radionuclides were deposited on the ground by dry and/or wet atmospheric deposition through rain, fog, or snow, depending on the meteorological conditions [2,3]. These two radionuclides, together with cesium-134 (^134^Cs), made the largest contribution by far in terms of public exposure [2]. Thus, the Japanese government, municipality and private companies have carried out the environmental and individual radiation monitoring including the external and internal exposure doses to confirm the radiation level affected areas by the FDNPS accident [4,5,6]. From these monitoring results, it is confirmed that artificial radionuclides with a relatively long half-life such as ^134^Cs (half-life: 2.1 y) and ^137^Cs (half-life: 30 y) still exist in the environmental samples including soils and plants in areas around the FDNPS [4,5,6].

During the eight years since the FDNPS accident, the levels of environmental radioactivity have been decreasing because of the natural decay of the radionuclides, meteorological conditions (weathering), and decontamination by the Japanese government and municipality including Tomioka Town [7,8,9,10]. The efforts of the Japanese government to reduce the estimated annual exposure dose rate to less than 20 mSv/y in the areas with an estimated annual exposure dose rate greater than 20 mSv/y (the restricted residence and difficult-to-return zones), and to reduce the estimated annual exposure dose rate closer to 1 mSv/y in the areas with an estimated annual exposure dose rate of less than 20 mSv/y (the evacuation order cancellation preparation zone and the evacuation order-lifted areas) are still ongoing; this is being done with the cooperation of local authorities and inhabitants through the implementation of effective decontamination work, which is being carried out according to the recommendations of the International Commission on Radiological Protection [4,11,12]. However, it is still necessary to evaluate the long-term behavior and exposure risk of radiocesium in the environmental samples such as soils by the radiation monitoring.

Tomioka town is located within a 20-km radius of the FDNPS. On 1 April 2017, with the Act on Special Measures Concerning Nuclear Emergency Preparedness, the Japanese government declared that residents who lived in approximately 88% of the gross area of the Tomioka town could return to their homes because the air dose rates were at low levels (estimated doses were expected to be less than 20 mSv/y) [4,5]. Although 1.5 years have passed since this “declaration of return,” as of 1 October 2018, there were 12,341 evacuees of the Tomioka town, 2627 (21.3%) of whom still currently live outside of Fukushima Prefecture, 9714 (78.7%) living somewhere in Fukushima Prefecture, and the rate of residents who have returned home in Tomioka town is still extremely low, at 791 (6.4%) [6]. The reason for this limited number is thought to be anxiety regarding exposure to radiation derived from the accident [13,14]. In fact, the “difficult-to-return” zone, where the integrated dose rates are over 50 mSv/y, represents approximately 12% of the gross area of the Tomioka town; therefore, the risk of external and internal exposure while residents perform activities of daily living (ADL) remains a particular concern, and some means to reassure the public safety are required [4].

**Figure 1:**
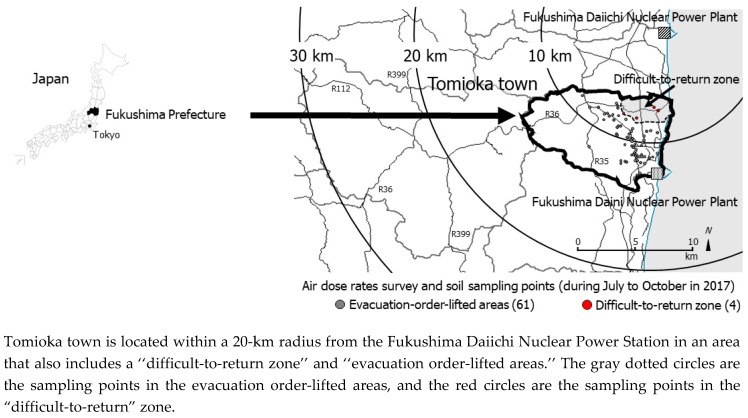
Location of Tomioka town, Fukushima Prefecture.

Our previous reports showed that the external and internal exposure dose rates among residents who had returned to Kawauchi village, which is adjacent to Tomioka town, were limited [15] (Figure 1). Nevertheless, long-term environmental monitoring, as well as efforts such as further decontamination and food monitoring, should continue around the FDNPS [16,17,18,19], including the Tomioka town. Especially, the external exposure risk on the livelihood zone of residents (returner’s living space) such as housing sites is not evaluated unlike the data which the municipal government including the national and municipality government have reported by literature, database and website. Therefore, in the present study, to evaluate the amount of environmental contamination and calculate the contributory external radiation exposure doses of residents who had already returned or who planned to return in the future, we measured air dose rates and analyzed the concentrations of artificial radionuclides in soil samples collected in the residential areas of the Tomioka town using gamma spectrometry (Figure 1).

## 2. Materials and Methods

### 2.1. Sampling Points

The FDNPS (37°25’ N, 141°02’ E) is located on the east coast of Honshu Island, approximately 200 km northeast of Tokyo. Tomioka town (public office: 37°20’ N, 141°0’ E) is located 8.5 km south of the FDNPS. In the present study, we measured air dose rates and collected soils from 65 sampling points in 45 residential areas where residents had returned home and near 20 assembly halls in Tomioka town between 11 July and 25 October 2017 (Figure 1).

### 2.2. Measurement of Air Dose Rates and Radionuclides

In the present study, the air dose rates were monitored in air 1 m above the ground at all sampling points using a NaI(Tl) scintillation survey meter (TCS-172B, Hitachi-Aloka Medical, Ltd., Tokyo, Japan), which can measure gamma rays (50 keV-3 MeV, ambient dose equivalent rate at 1cm depth: 0.00–30.0 μSv/h). We measured air dose rates with a time constant of 10. In the evacuation order-lifted areas, an additional radiation dose (radiocesium) including the natural dose was estimated using the following formula:

*A_ext_* (mSv/y) *=* [(*C_int_* − 0.04 μSv/h)∙*16*h + (*C_ext_* − 0.04 μSv/h)∙8 h]∙365 d∙0.001 (1)

where *C_int_* is the indoor air dose rate (μSv/h) and *C_ext_* is the outdoor air dose rate (μSv/h). The fixed number (0.04 μSv/h) in the formula is defined as the natural dose in Japan, and 16 h and 8 h are defined as representing the indoor and outdoor ADL, respectively. This calculation was based on the method described by the Ministry of the Environment [8]. According to the monitoring information by the national and local authorities, the prevalent dose-forming artificial radionuclides from various samples have been mainly ^134^Cs and/or ^137^Cs in offsite areas around the FDNPS. [4,5].

At the same time, to evaluate the vertical distribution and external radiation exposure, 130 samples (65 sites × 2) of surface soil (0–5 and 5–10 cm below the surface) were collected at Tomioka town from July to October 2017. Soil sampling was carried out at all sampling sites using a core sampling technique (two core samples for each point). The size of the soil samples was 18.2 cm^2^ (diameter of 4.8 cm) and the density of the soil layers (0–10 cm) ranged from 0.31 to 2.3 g/cm^3^ (dry). The mass of the soil samples collected in each area ranged from 24.5 to 284 g. After collection, all samples were dried for 24 h in a fixed temperature dryer (105 °C). Next, the samples were sieved for pebbles and organic materials (>2 mm). After preparation, the samples were placed in plastic containers made of polypropylene and analyzed using a high purity germanium detector (ORTEC^®^ GMX series, Ortec International Inc., Oak Ridge, TN, USA) coupled to a multi-channel analyzer (MCA7600, Seiko EG&G Co., Ltd., Chiba, Japan) for 3600–36,000 s. We set the measuring time to detect objective radionuclide levels. The target gamma ray peaks used for the measurements were 604.66 keV for ^134^Cs (half-life: 2.1 y) and 661.64 keV for ^137^Cs (half-life: 30 y). Decay corrections were made based on the sampling date. The detector efficiency calibration for different measurement geometries including the density and thickness of samples was performed using mixed activity standard volume sources (Japan Radioisotope Association, Tokyo, Japan); the relative detection efficiency of this instrument was 33.04%. Sample collection, processing, and analysis were executed in accordance with standard methods of the radioactivity measurement authorized by the Ministry of Education, Culture, Sports, Science, and Technology, Japan [20]. All measurements were performed at Nagasaki University, Nagasaki, Japan. The obtained data are expressed as average, range (minimum–maximum) and medians.

### 2.3. Effective Dose Rate

After the measurements, external effective dose rates (μSv/h and mSv/y) from soil samples were estimated from artificial radionuclide concentrations using the following formula:

*H_ext_* = *C*∙*D_ext_*∙*f*∙*s* (2)

where *C* is the activity concentration (median) of detected artificial radionuclides (^134^Cs and ^137^Cs) (kBq/m^2^; estimated from the radiocesium concentration in Bq/kg, including soil particles (<2 mm) and collected surface soil (0.00182 m^2^)), *D_ext_* is the dose conversion coefficient reported as the kerma-rate in air at 1 m above the ground per unit activity per unit area ((μGy/h)/(kBq/m^2^)), supposing that the air-kerma rate and the absorbed dose rate in air were the same value, for radiocesium with the relaxation mass per unit area (β: g/cm^2^) set to 10 (5-20 y) because more than eight years had passed since the FDNPS accident (1.95×10^-3^ (μGy/h)/(kBq/m^2^) for ^134^Cs and 7.55×10^−4^ (μGy/h)/(kBq/m^2^) for ^137^Cs, ICRU 1994) [21], *f* is the unit conversion coefficient (0.7 Sv/Gy for the effective dose rate in the body per unit absorbed dose rate in air) [22], and *s* is the decrease in the coefficient by a shielding factor against exposure to gamma rays from a sample at 1 m above the ground (0.7 under the condition of usual land) [23]. These calculations were based on the method described in our previous study [15,16,24].

### 2.4. Ethics Statement

The present study was approved by the ethics committee of Nagasaki University Graduate School of Biomedical Sciences (project registration number: 17030212), and written informed consent was obtained from the owners of the land containing all sampling points.

## 3. Results

The air dose rates in Tomioka town are shown in Table 1. In the evacuation order-lifted areas, the median air dose rates inside the homes of the residents who had returned were 0.20 [0.086–0.37] μSv/h indoors, 0.26 [0.088–0.68] μSv/h outdoors (in front of the entrance), and 0.34 [0.14–1.3] μSv/h in the backyard. The annual estimated doses were 1.7 mSv/y indoors, 2.3 mSv/y outdoors, and 3.0 mSv/y in the backyard. Therefore, an additional radiation exposure dose of 1.6 mSv/y was estimated by the formula (1). On the other hand, in the difficult-to-return zone, the median air dose rates were 2.3 [1.1–2.9] μSv/h outdoors and 2.1 [1.8–2.4] μSv/h in the backyard. The annual estimated doses were 20 mSv/y outdoors and 18 mSv/y in the backyard.

**Figure 2:**
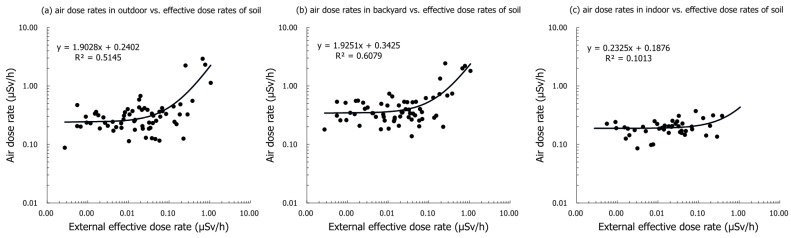
Relationship between estimated external effective dose rates from surface soil and air dose rates in Tomioka town. (**a**) Air dose rates in outdoor (entrance) vs., (**b**) air dose rates in outdoors (backyard) vs. and (**c**) air dose rates in indoor (entrance) vs. effective dose rates of soil samples. The external effective dose rates from soil samples were estimated using a high purity germanium detector (only radiocesium). Air dose rates were measured at the sampling points using a NaI (Tl) scintillation survey meter (natural dose rates including radiocesium).

The distribution of detected artificial radionuclides (radiocesium) and external effective dose rates from the surface soil due to radiocesium and the radionuclide ratios (^134^Cs/^137^Cs in Bq/kg [dry]) in Tomioka town are shown in Table 2. The dose-forming artificial radionuclides ^134^Cs and ^137^Cs were prevalent in all samples. In the evacuation order-lifted areas, the radiocesium concentrations inside the homes of the residents who had returned were 238 (8.0–6063) (0–5 cm) Bq/kg (dry) and 334 (3.7–5803) (5–10 cm) Bq/kg (dry) for ^134^Cs, and 1784 (34–45,331) (0–5 cm) Bq/kg (dry) and 2093 (28–48,911) (5–10 cm) Bq/kg (dry) for ^137^Cs. The external effective dose rates from the surface soil (0–5 cm) were estimated as 0.17 mSv/y. On the other hand, in the difficult-to-return zone, the radiocesium concentrations inside the assembly halls were 8025 (3317–18,552) (0–5 cm) Bq/kg (dry) and 6633 (4654–9034) (5–10 cm) Bq/kg (dry) for ^134^Cs, and 62,131 (25,559–141,209) (0–5 cm) Bq/kg-dry and 51,840 (36,317–69,377) (5–10 cm) Bq/kg-dry for ^137^Cs. The external effective dose rates from the surface soil (0–5 cm) were estimated as 6.4 mSv/y. In the evacuation order-lifted areas, the radiocesium concentrations were lower in the surface soil samples (0–5 cm) than in the lower layers (5–10 cm), whereas in the difficult-to-return zone, the radiocesium concentrations were higher in the surface soil samples (0–5 cm) than in the lower layers (5–10 cm). Therefore, in the difficult-to-return zone, there was still an accumulation of radiocesium in the surface layer. In the present study, the concentrations of radiocesium exceeded 8000 Bq/kg (dry), which is the standard value for storing decontamination waste according to the Japanese guidelines, at some (17) sampling points, and the median radiocesium values (^134^Cs/^137^Cs ratios) in the soil samples were 0.13 (0.093–0.18) at the time of sampling, regardless of whether they were in the difficult-to-return zone [25]. Moreover, the effective dose rates from the air dose rates in outdoors and soil samples in Tomioka town showed a positive relationship (r = 0.51 and 0.61, Figure 2).

**Figure 3:**
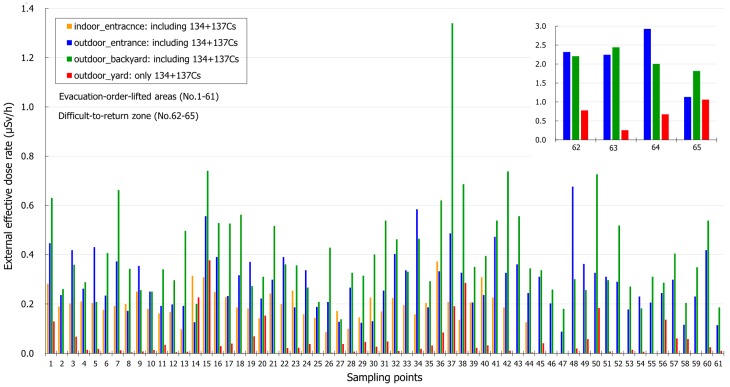
External exposure doses immediately after the cancellation of restriction in Tomioka Town. The effective external doses in residents living space within housing sites during September to October in 2017. Yellow, blue and green squares show the natural dose rates including radiocesium. Red square shows the external effective dose rates from soil samples (only radiocesium).

The effective external doses in the living space of residents within housing sites immediately after the cancellation of the restriction in Tomioka Town (during September to October in 2017) are shown in Figure 3. In the present study, the external exposure doses including radiocesium were mainly higher in the backyard than outdoors in front of the entrance. Naturally, the external exposure doses including radiocesium were higher in outdoors than indoors by the shielding effectiveness of their house. Moreover, the external exposure doses due to radiocesium in soil samples were sufficiently low level.

## 4. Discussion

In the present study, the artificial radionuclides ^134^Cs and ^137^Cs were detected in all samples from Tomioka town by gamma spectrometry, and the ^134^Cs/^137^Cs values of these samples were around 0.13, which is thought to be the consequence of the relatively early decay of ^134^Cs (median: 238 (8.0−6063) Bq/kg (dry) for ^134^Cs and 1784 (34−45,331) Bq/kg (dry) for ^137^Cs in the upper layer (0−5 cm); see Table 2). Immediately after the accident, the ^134^Cs/^137^Cs values were reported as 0.9 in areas to the south and southwest of the FDNPS, which were higher than those observed around the Chernobyl Nuclear Power Plant [26,27]. Eight years have passed since the FDNPS accident, and it was confirmed that the ^134^Cs/^137^Cs values have been decreasing because of the natural decay of ^134^Cs (half-life: 2.1 y). Therefore, in the present study, the radiocesium (the ^134^Cs/^137^Cs values: 0.13 (median)) detected in these samples was obviously derived from the FDNPS accident.

In the present study, air dose rates were higher in the backyard (where trees and plants such as cedar were growing) than outdoors in front of the entrance to homes in evacuation order-lifted areas (0.34 (0.14−1.3) μSv/h in the backyard vs. 0.26 (0.088–0.68) μSv/h outdoors and 0.20 (0.086–0.37) μSv/h indoors; Table 1). In the difficult-to-return zone, the outdoor air dose rates (in the backyard and in front of the entrance) were nearly equivalent, and the samples were still contaminated (2.1 (1.8−2.4) μSv/h in the backyard vs. 2.3 (1.1–2.9) μSv/h outdoors; Table 1). Also, gaps were observed between air dose rates and estimated external effective dose rates. The external effective dose rate in the evacuation-order-lifted areas were estimated at 10 times higher than the external estimated effective dose rates from radiocesium in soil samples (Table 1 and Table 2). However, because a positive relationship (r = 0.61) was observed between air dose rates in the backyard and estimated effective dose rates from surface soil samples, the findings of the present study suggest that the environmental radiation dose was mainly derived from surface soil and areas around vegetation, including fallen leaves (Figure 2). Following the FDNPS accident, the residential areas, farmlands, forests (the close to residential areas; <20 m), and roads within the evacuation order areas around the FDNPS were extensively decontaminated by suitable methods, and decontamination of the entire area, excluding the difficult-to-return zones, was completed on 19 March 2018 [25]. However, the decontamination effect of areas around vegetation including fallen leaves may be limited. Some reports have suggested that radiocesium accumulates in various forest environments through the forest biota [24,28,29,30,31]. The detection of high ^137^Cs concentrations in evergreen cedar needles after the accident indicated that the canopy interception of atmospherically deposited ^137^Cs, and the existence of high ^137^Cs activity in newly developed foliage during the six years after the accident, particularly in the leaves of Japanese konara oak (*Quercus serrata*) seedlings in an abandoned coppice forest, suggests translocation and efficient recycling of ^137^Cs within the trees [32]. On the other hand, the effective dose rates from the air dose rates in indoors and soil samples in Tomioka town showed a negative relationship (r = 0.10, Figure 2). The shielding effect by houses was comparatively high because there were a lot of new houses with high airtightness in areas of the cancellation of restriction in the Tomioka Town (shielding factor = 0.77, Table 1) [22,23]. Actually, the additional radiation exposure dose was estimated at low level (1.6 mSv/y) [33].

The Japanese and local government aim to reduce the estimated annual exposure dose rate to 1 mSv/y as early as possible and continue with further reductions in residential areas. In the present study, the current contamination levels due to radiocesium were extremely different in both areas; low (0.17 mSv/y) in the evacuation order-lifted areas and relatively high (6.4 mSv/y) in the difficult-to-return zone in Tomioka town (Table 2). In the eight years that have passed since the FDNPS accident, the estimated external exposure levels in the evacuation order-lifted areas have decreased because of decontamination and the decay of artificial radionuclides; however, further remediation of soil contaminated with artificial radionuclides in the difficult-to-return zone, which is a crucial social responsibility in Japan and internationally, is still needed. Based on the current findings regarding radiocesium concentrations and effective dose rates, decreases in external exposure doses are the evidence that evacuees from the Tomioka town may return (Figure 3). Especially, the concentrations of detected radiocesium in surface soils were low during two layers (0–5 cm and 5–10 cm) in the evacuation order-lifted areas of Tomioka town, and the current levels of environmental contamination around homes in this area of Tomioka town are extremely low. Conversely, in the difficult-to-return zone, the concentrations of detected radiocesium were higher in surface soil samples (0–5 cm) than in lower layers (5–10 cm), because in this area, effective decontamination of the strong absorption of radiocesium by soil particles is not progressing smoothly (Table 2). Most ^137^Cs accumulated within 1.5 years after the FDNPS accident and ^137^Cs continued to be retained in the upper mineral soil layer (0–5 cm) [34]. Absorption of ^137^Cs appears to be the primary process regulating the ^137^Cs distribution in the soil profiles (vertical distribution) over five years of monitoring after contamination [34]. In other words, these findings suggest that environmental contamination and the effective dose rates on the ground in the evacuation order-lifted areas will be decreased by decontamination procedures, such as the removal of surface soil [35] (Figure 3). In addition, the decontamination of residential areas, farmlands, forests, and roads is being carried out by suitable methods [35]. Reconstruction projects have already started in the area of the difficult-to-return zone in coordination with relevant ministries and agencies such as the Ministry of the Environment and Reconstruction Agency [36]. In Tomioka town, the pre-decontamination of the difficult-to-return zone has been carried out [37]. The investigation of the Ministry of the Environment and our research by the car-survey report the effectiveness of the pre-decontamination in the difficult-to-return zone (data not shown). It is expected that evacuation orders will be lifted for wider areas in the difficult-to-return zone in the near future.

This study did have several limitations. First, the number of soil sampling points were relatively limited, especially in the difficult-to-return zone (*n* = 4), because the size of the sample collection was small under the serious and emergent conditions of the FDNPS accident. Although we researched in the residential area as much as possible for the return to home in the future, further investigation with detailed conditions is needed while confirming the change of radiation doses by the decontamination work.

Second, other types of evidence such as the internal exposure doses, infrastructure repairs and support services also play roles in the decision to return. Especially, agricultural activities and the countryside (*satoyama*) cultural practice of ingesting edible wild plants (*sansai*) and mushrooms are being carried out carefully based on the guidelines by the Nuclear Emergency Response Headquarters of the Japanese government, accompanied by the ongoing decontamination of farmlands and radiation monitoring [5,38]. Edible wild plants and mushrooms are well-known as accumulators of radiocesium [16,18,19,39,40,41,42]. Currently, the shipment of agricultural products in Japan is determined based on regulations outlined by the Japanese government [43]. In Tomioka town, the monitoring system for local foods by using the nondestructive equipment for detecting radiocesium such as the NaI spectrometer is an effective tool to avoid unnecessary radiation exposure since February 2018. Further investigations on external and internal effective doses, are needed [44,45].

## 5. Conclusions

In the present study, we evaluated environmental contamination and contributions from the external exposure due to radiocesium in Tomioka town near the FDNPS. Based on the current findings regarding radiocesium concentrations and effective dose rates, we confirmed that current levels are decreasing sufficiently, especially in the evacuation order-lifted areas located within a 20-km radius from the FDNPS and decontaminated rapidly, even though a certain amount of radiocesium derived from the accident was detected in soil samples in these areas. Thus, decreases in external exposure doses are the evidence that that evacuees from Tomioka town may return with the long-term follow-up, as well as environmental monitoring and countermeasures, such as further decontamination and restrictions on the intake of local foods (edible wild plants and mushrooms) that can cause unnecessary radiation exposure, and physical and mental support [35,36,37]. The case of Tomioka town is expected to be the first reconstruction model for evaluating environmental contamination and radiation exposure dose rates due to artificial radionuclides, including areas such as the difficult-to-return zone near the FDNPS.

## Figures and Tables

**Table 1:** Air dose rates around residences and assembly halls in Tomioka town, Fukushima Prefecture, during September to October 2017.

Points		Air Dose Rate in μSv/h			External Effective Dose Rate in mSv/y	Shielding Factor
		Average	Range	Median		
Evacuation order-lifted areas (*n* = 61) *^a^*	Indoors	0.20 ± 0.058 *^c^*	0.086–0.37 *^d^*	0.20 (0.28) *^e^*	1.7 *^f^*	0.77 *^g^*
	Outdoors	0.29 ± 0.12	0.088–0.68	0.26 (0.43)	2.3	
	Backyard	0.40 ± 0.19	0.14–1.34	0.34 (0.63)	3.0	
Difficult-to-return zone (*n* = 4) *^b^*	Outdoors	2.2 ± 0.65	1.1–2.9	2.3 (2.7)	20	
	Backyard	2.1 ± 0.23	1.8–2.4	2.1 (2.4)	18	

*^a^* residences (*n* = 45) and assembly halls (*n* = 16). *^b^* assembly halls (*n* = 4). *^c^* mean ± S.D. *^d^* minimum-maximum. *^e^* parentheses show 90th percentile. *^f^* median × 24h × 365d × 0.001. *^g^* shielding factor of air dose rates ratio (indoors/outdoors).

**Table 2:** Distribution of radiocesium in soil samples in Tomioka town, Fukushima Prefecture.

Points		Radiocesium Concentration in Bq/kg (dry) *^a^*						External Effective Dose Rate in mSv/y	Radionuclide Ratio in ^134^Cs/^137^Cs
	depth	Average		Range		Median			
		^134^Cs (2.1 y)	^137^Cs (30 y)	^134^Cs (2.1 y)	^137^Cs (30 y)	^134^Cs (2.1 y)	^137^Cs (30 y)		
Evacuation-order-lifted areas (*n* = 61) *^a^*	0–5 cm	694.3 ± 1137 *^c^*	4996 ± 8421	8.0–6063 *^d^*	34–45,331	238 (1950) *^e^*	1784 (12,966)	0.17 *^f^*	0.13 (0.14) *^g^*
	5–10 cm	750.0 ± 1035	5585 ± 8163	3.7–5803	28–48,911	334 (2016)	2093 (15,209)		0.13 (0.14)
Difficult-to-return zone (*n* = 4) *^b^*	0–5 cm	9480 ± 5708	72,757 ± 43,211	3317–18,552	25,559–141,209	8025 (15,906)	62,131 (121,336)	6.4	0.13 (0.13)
	5–10 cm	6739 ± 2067	52,343 ± 15,690	4654–9034	36,317–69,377	6633 (8893)	51,840 (68,551)		0.13 (0.14)

*^a^* Residences (*n* = 45) and assembly halls (*n* = 16). *^b^* Assembly halls (*n* = 4). *^c^* mean ± S.D. *^d^* minimum-maximum. *^e^* parentheses show 90th percentile. *^f^* calculated by the formula (2). *^g^* median (90th percentile).
